# Abdominal pain in a young man revealing an infected perigastric cystic lymphangioma

**DOI:** 10.1055/a-2081-7882

**Published:** 2023-05-15

**Authors:** Pierre Mayer, Emanuele Felli, Patrick Pessaux, Jérôme Huppertz, François Habersetzer, Lucile Héroin, Guillaume Philouze

**Affiliations:** 1 Department of Hepatology and Gastroenterology, Pôle Hépato-digestif, Nouvel Hôpital Civil, Hôpitaux Universitaires de Strasbourg (HUS), Strasbourg, France; 2 IHU-Strasbourg (Institut Hospitalo-Universitaire), Strasbourg, France; 3 Liver Transplant and Surgery Department, Hôpital Trousseau, Tours, France; 4 Inserm U1110, Institute for Viral and Liver Diseases, LabEx HepSYS, University of Strasbourg, Faculty of Medicine, Strasbourg, France; 5 Department of Visceral and Digestive Surgery Pôle Hépato-digestif, Nouvel Hôpital Civil, Hôpitaux Universitaires de Strasbourg (HUS), Strasbourg, France; 6 Department of Gastroenterology and Hepatology, Clinique Sainte Barbe, Strasbourg, France

Abdominal cystic lymphangiomas are a rare pathology of childhood. They are lymphatic malformations that develop in the retroperitoneum or mesenteric space 1 2 . They are most often asymptomatic and are revealed during an acute complication. When they are symptomatic, surgical resection is the rule 3 .

We report the case of a 17-year-old patient with no medical history referred for abdominal pain and fever. Biologically, we only found inflammatory syndrome. The abdominal computed tomography (CT) scan showed a collection under the stomach and above the pancreatic tail. There was a fatty infiltration suggesting inflammatory involvement. Magnetic resonance imaging of the abdomen revealed a lesion measuring 64 × 39 mm of retro-gastric topography. This lesion presented regular sharp contours. There was intense and homogeneous contrast of the shell, with a heterogeneous upper portion that appeared almost adherent to the gastric wall ( Fig. 1 ). We performed endoscopic ultrasonography (EUS), which found a retroperitoneal cyst located between the splenic hilum, the left adrenal gland, the left kidney, and the tail of the pancreas. The lesion presented an abscessed appearance with a vascularized wall ( Fig. 2 ). There was no intra- or retroperitoneal adenopathy ( Video 1 ). Antibiotic therapy was started for 8 days. The diagnosis of an infected retro-gastric cystic lymphangioma was retained. Under antibiotic therapy, the evolution was rapidly favorable, and surgical indication was retained. An abdominal CT scan performed 4 months later showed a reduction of the homogeneous retro-gastric lesion ( Fig. 3 ). A laparoscopic resection was performed with monobloc retro-gastric lymph node dissection. The postoperative course was simple and anatomopathological findings confirmed the diagnosis 4 5 .

**Fig. 1:**
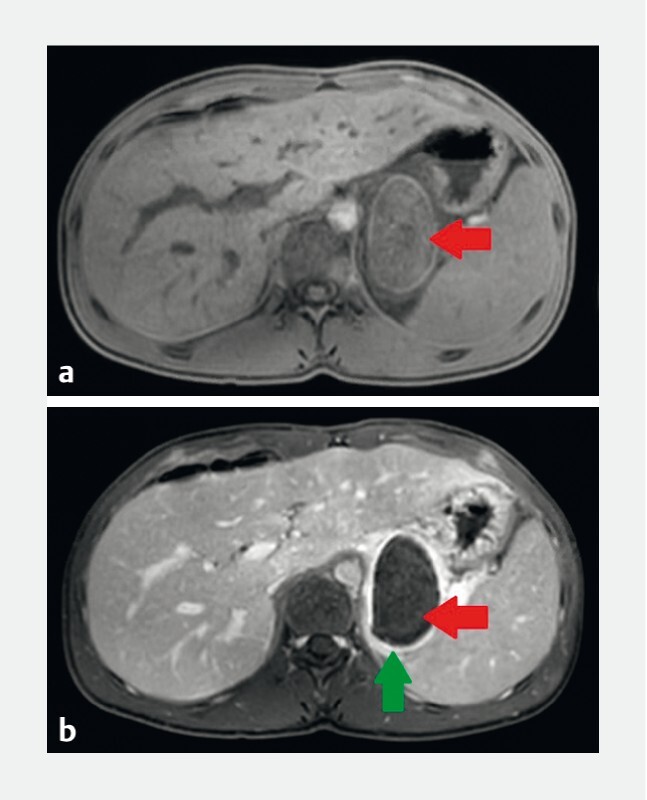
Abdominal magnetic resonance imaging showing a retro-gastric cystic lymphangioma. **a** Cystic lymphangioma located behind the stomach (red arrow) with homogeneous content in T1 sequence. **b** Cystic lymphangioma located behind the stomach (red arrow) with significant wall enhancement after contrast injection (green arrow).

**Fig. 2:**
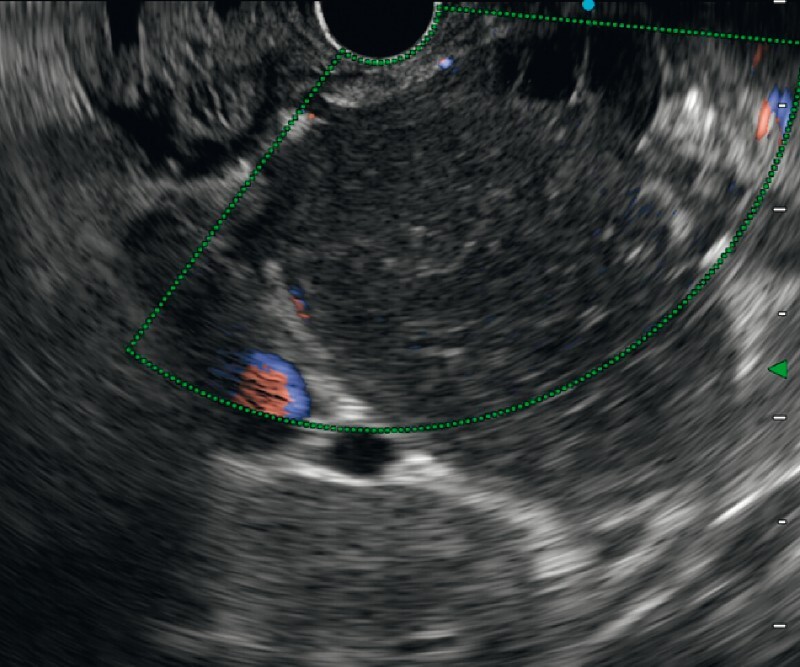
Endoscopic ultrasound appearance of cystic lymphangioma with vascularized walls.

**Video 1** Endoscopic ultrasound confirming the diagnosis of cystic lymphangioma with argument for infection leading to its surgical resection.


**Fig. 3:**
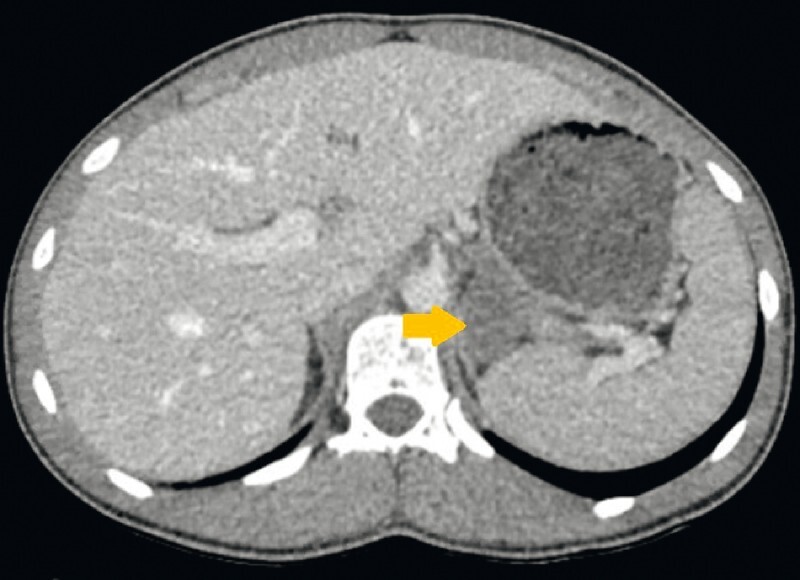
Abdominal computed tomography scan 4 months after the infectious episode, showing a decrease in size of the retro-gastric cystic lymphangioma (yellow arrow).

Most symptomatic cystic lymphangiomas are revealed by abdominal pain, but no case of infection has ever been described. EUS, in addition to other imaging techniques, allows the diagnosis, especially if the latter are insufficient for diagnosis. Surgical treatment remains the reference for this type of condition.

Endoscopy_UCTN_Code_CCL_1AB_2AG_3AC

## Notes

Endoscopy E-Videos

https://eref.thieme.de/e-videos
*E-Videos* is an open access online section of the journal *Endoscopy* , reporting on interesting cases and new techniques in gastroenterological endoscopy. All papers include a high-quality video and are published with a Creative Commons CC-BY license. Endoscopy E-Videos qualify for HINARI discounts and waivers and eligibility is automatically checked during the submission process. We grant 100% waivers to articles whose corresponding authors are based in Group A countries and 50% waivers to those who are based in Group B countries as classified by Research4Life (see: https://www.research4life.org/access/eligibility/ ). This section has its own submission website at https://mc.manuscriptcentral.com/e-videos
